# Coping with the COVID-19 Pandemic: Comparative Case Study of Coping and Resilience in Children From Different Educational Contexts in Colombia

**DOI:** 10.5334/cie.56

**Published:** 2023-01-16

**Authors:** Maria Fernanda Gonzalez-Puerto, Ingrid Anzelin, Sebastian Calixto, Roberto Alvira

**Affiliations:** 1 School of Education, Universidad de La Sabana, Chía, Colombia; 2 Faculty of Law and Political Science, National University, Bogota, Colombia

**Keywords:** resilience, coping, pandemic, social educational context, children

## Abstract

In 2020, humanity experienced one of the most complex situations in history: The COVID-19 pandemic, which caused significant social, economic, and educational consequences. Nevertheless, countries and people generally survived. Why? Resilience and the ability to cope are fundamental elements in human, community, and national survival. This study compared the situations experienced by six children from different social and educational backgrounds in Colombia during the COVID 19 pandemic using a collective analysis of cases. Interviews with children’s families, as well as observations of the participants and a narrative instrument from the BASIC Ph resiliency model (Lahad, 2016) are used to describe the context, the promoting factors of resilience, and the so-called coping “channels” of each case. Results showed that (a) the difficulties perceived by families during the pandemic were different from those perceived by the children, and (b) the predominant coping channels had an important relationship with factors that promote resilience. Finally, although it is not clear from the present study whether the children developed resilience, they exhibited factors that determine their future development.

Resilience and coping are fundamental elements in human, family, and community development. Throughout life, people experience complex situations that test all their resources and coping skills. One such situation was the COVID-19 pandemic, a health crisis that affected people worldwide in one way or another and demonstrated the importance of developing skills that promote positive coping and resilience.

Specifically, the pandemic generated different types of affective and educational consequences depending on the social, family, and individual context of each person. Even though adults are more likely to be infected with the virus, children suffer more psychological repercussions such as behavioral problems and anxiety due to isolation (Capurso et al., 2020).

This study describes and compares the coping and resilience processes of six children between 6 and 8 years old from different geographical and educational contexts in Colombia in response to the educational changes resulting from the COVID-19 pandemic. Specifically, we looked at the educational, social, family, cultural and personal contexts of each case; the difficulties experienced during the pandemic; the so-called coping “channels” of the BASIC Ph model (Lahad et al., 2013); the factors promoting resilience; and comparison of the findings according to the educational context of each case in order to answer the question: What were the coping and resilience processes of six children between 6 and 8 years old, from different geographical and educational contexts in Colombia, during the COVID-19 pandemic?

## Coping

Coping is defined as the process of dealing with stress derived from individual efforts to maintain control of a situation (Lazarus & Folkman, 1984). In other words, it refers to the action of dealing with a threats, dangers, stressors, or responsibility through thoughts, feelings, and actions that reduce the adverse characteristics of the situation (Ortuzar & Di Meglio, 2008).

Therefore, coping refers to the different individual emotional, cognitive, and behavioral responses that a person uses to manage stress (Skinner & Zimmer, 2006). But what is stress? In his coping model, Lazarus defined stress as situations when an individual’s available resources are exceeded by the demands of the environment (Lazarus & Folkman, 1984). However, depending on the context of the person, a given situation may or may not be stressful. For this reason, the types of stressors are culturally defined by the beliefs, values, agreements, behaviors, and resources of the individual and their context (Aldwin, 2004).

Nevertheless, it is important to note that coping is not synonymous with resilience; these concepts are dependent on each other. Moreover, it has been observed that positive coping leads to adaptation to the situation (Kumpfer, 1999), whereas resilient people tend to use active or positive strategies of a behavioral or cognitive type in order to avoid stress (Anthony, 2008).

## Resilience

Resilience, on the other hand, is a complex concept with multiple definitions. Etymologically, the word comes from the Latin verb “resilire,” which translates into English as “bounce” or “jump back” (Kotliarenco et al., 1997). The present research focused on the concept of resilience in the field of social sciences, where three schools of research on resilience originally emerged in two different territories: the Anglo-Saxon school and the European school (Forés & Grané, 2012). Other important models of resilience have emerged in the Middle East and Latin America.

Specifically, within the Anglo-Saxon school, Rutter (as cited in Feder, 2018) first used the term “resilience” in psychology in 1978 to explain the ability of human beings to protect themselves from risks and adverse situations through mechanisms that promote self-esteem and interpersonal relationships.

For example, Rutter (as cited in Shean, 2015) clarified that low-level risks, or challenges, are essential for the development of resilience-promoting protective factors such as planning, self-control, and determination. Similarly, Werner (as cited in Shean, 2015) found that the development of resilience depends on adequate psychosocial interaction and individual, family, and community factors at each stage of development.

Along with Rutter and Werner, Garmnezy (as cited in Shean, 2015) conducted the Competence Project out of his concern for the developmental disadvantages of children in poverty. According to Garmnezy, resilience is the ability to recover and maintain adaptive behavior in the face of a situation that affects one’s emotional balance. Like Werner and Rutter, Garmenzy held an ecological view of resilience based on the development of individual, family, and contextual factors.

In addition to the theories and models suggested by researchers within the Anglo-Saxon school of thought, the study of resilience is a global phenomenon with context-specific characteristics. For example, some of the authors within the European school include Cyrulnik (Aprendamos Juntos BBVA, 2018) and Vanistendael (1994). In this school, resilience is defined as a process of overcoming adversity and trauma; that is, the personal narrative framework is important in considering the person as the active protagonist of their resilient action (Forés & Grané, 2012).

Ungar, a Canadian researcher, realized that there was little rigorous research on resilience outside the Western context and, therefore, conducted a mixed-research study in 14 communities on 5 continents called the International Resilience Project (IRP). Ungar (2008) defined resilience as a multidimensional construct whose characteristics are specific to the relationship between individuals and their community.

The study of resilience in Latin America is characterized by a wide scope of research action, but with little impact at the international level. Indeed, research on resilience in Latin America has focused on community resilience; more specifically, on identification of the social conditions, group relations, cultural manifestations, and community values that form community resilience (Forés & Grané, 2012).

In Colombia, for example, resilience is indispensable due to the various psychosocial risk factors that may affect the physical and mental health of the population, such as violence and poverty, among others. As a result, there is a particular interest in studying resilience in childhood, identifying risk factors and the protective factors that promote resilience (Duarte, 2016).

### Resilience Factors

To facilitate an understanding of the categories of analysis of this study, the resilience factors will be analyzed by levels or subsystems as proposed by Wolin (as cited in Melillo & Suárez, 2002), who defined the pillars of resilience as those attributes of people considered resilient that include creativity, humor, morality, introspection, the ability to relate to others, independence, and initiative. To these pillars were later taken by Suárez, who added social competence, problem solving, autonomy, and positive expectations for the future (Melillo & Suárez, 2002).

Not all factors involved in developing resilience come from the individual. In the case of the affective bonds that children have with adults – known as “attachment” – these are determining components of resilience. In other words, “resilience is not an innate individual attribute, independent from the environment but emerges from the child’s relationship with his or her fundamental environment: the human environment” (Barudy & Dantagnan, 2011, p. 17).

Consequently, the family is a source of resilience because, family relationships determine how the child will react to stressful situations. Therefore, if children receive affection and support at an early stage, they will probably become autonomous persons and develop resilience (Aparicio et al., 2019). Moreover, a study conducted by the International Children’s Center found that children born to resilient mothers also developed resilience (Simpson, 2017).

Apart from this, socio-educational scenarios facilitate the development of resilience by representing a socialization and learning environment (Forés & Grané, 2012). That is, the school or college becomes a context for building resilience by allowing the development of children’s autonomy and facilitating the construction of qualities with others that lead to resilience (Uriarte, 2006).

Certain school factors promote resilience, such as affectionate attention from teachers, positive social relationships, achievement motivation (Werner & Smith, 1982), positive evaluation by teachers (Radke-Yarrow & Brown, 1993), a sense of belonging to the school, shared educational goals, a non-conflictive school environment, good communication between the family and the school (Uriarte, 2006), and educational interventions that include trauma-processing activities (Capurso et al., 2020).

In addition to the family and school environment, the community can also be resilient and affect the development of resilience in the individuals that make up the community. For this reason, some of the pillars of community resilience observed in Latin American communities include collective self-esteem, cultural identity, social humor, and state honesty (Melillo & Suárez, 2002).

## BASIC Ph Model

For the purposes of this research, the theoretical considerations of the BASIC Ph model will be discussed because it describes all possible coping and resilience responses considered by Lahad et al. (2013), who constructed an integrative and multifaceted model of resilience composed of the interaction between six dimensions or coping channels: Belief, Affect, Social, Imagination, Cognition, and Physical. According to this model, people have the capacity to act with one or more of the coping channels. However, depending on their life experiences, innate tendencies, or contextual influences, people tend to cope with stressful situations predominantly using one or two channels (Ajdukovic et al., 2014).

To gain a deeper understanding of the current state of knowledge about the topic under study, we conducted a literature review, which resulted in identification of certain factors and mechanisms that promote resilience in each of the categories of the BASIC Ph model. It is important to mention that a single factor does not always correspond to a single dimension but may be belong to one or more resilience channels (Lahad et al., 2013). For example, self-efficacy, self-esteem, and self-understanding were considered factors of the Belief and Cognition channels; the presence of an emotionally stable person during childhood and a healthy type of attachment were considered to belong to the Affect and Social dimensions; and easy temperament characteristics were considered as factors of the Social, Affect, and Physical channels, among many other findings (Lahad et al., 2013).

To assess coping channels, the Six-Part Story Method (6PSM) proposed by Lahad et al. (2013) was used. This instrument aims to assess the resilience and coping channels of children and adults through the creation of a story in response to six questions. It is based on psycholinguistics, a branch of psychology that focuses on the construction of stories to analyze the way in which people understand information and transmit their ideas (Lahad et al., 2013).

Both the 6PSM and the open-ended questions allow individuals to explore internal realities in a safe environment through the creation of a fictional character and situation (Dent-Brown, as cited in Vettraino, 2016). This approach is also supported by Cyrulnik and Anaut’s (2018) view of how music, movies, books, or works of art allow characters to become spokespersons who convey what people otherwise do not dare to tell.

## Research on Child Resilience During the COVID-19 Pandemic

The first studies conducted with children at the beginning of the pandemic found that the quarantine triggered behaviors such as distraction, irritability, and dependency. Specifically, among children between 3 and 6 years of age, there was a higher prevalence of dependent behaviors and fear that one of their family members might become infected by the virus (Yan et al., 2020).

As mentioned, several mechanisms may be used to cope with difficult situations, including resilience. For example, a survey conducted in Spain of the parents of children between 3 and 12 years of age found that the strategies used by children to overcome the crisis varied according to their age (Domínguez et al., 2020). Similarly, a study carried out in Chile asked parents or guardians of children between 4 and 18 years of age about the educational experiences of these minors during the pandemic. Findings showed large inequalities in terms of access to education; specifically, more than 10% of children had not had access to remote education and experienced a significant increase in emotions such as anxiety, fear, and listlessness (Mancilla et al., 2021).

Moreover, school re-entry interventions in Italy prepared teachers to implement activities that increased children’s awareness and confidence about themselves and their environment during their return to school after the isolation period (Capurso et al., 2020). This program used free play sessions, classroom discussions, art, and literature to provide scenarios for students to express their feelings and concerns, and to train them in the use of information about COVID-19 pandemic (Capurso et al., 2020).

## Method

**Figure 1:**
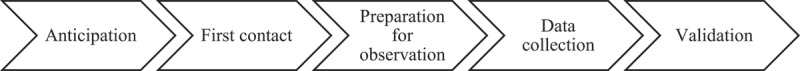
Stages of Methodology.

Considering that this study was using a qualitative approach, specifically a collective case-study design, we applied a five-step method in line with Stake (1998) (Figure 1). This approach was selected because qualitative studies aim to understand the way in which people perceive and experience a specific phenomenon through their meanings and interpretations (Hernández et al., 2019).

A case selection process was carried out based on specific criteria. Specifically, cases from greatly differing circumstances were selected, taking into consideration that the objective was to draw information from subjects in different contexts (Kazez, 2009) to study their processes of coping and resilience during the COVID-19 pandemic. Thus, the cases were selected based on the characteristics of their educational subsystem, their type of territoriality, and their alternative or diversity elements (Table 1).

**Table 1:** Selection Criteria for Cases.


CASE	SELECTION CRITERIA

Public urban school	Child between 6 and 8 years old living in a city in Colombia and attends a public school

Public rural school	Child between 6 and 8 years old living in a rural area of Colombia and attends a rural school

Private urban school	Child between 6 and 8 years old living in a city in Colombia and attends a private school

Ethnic School	Child between 6 and 8 years old living in an indigenous reservation in Colombia and attends an ethnic school

Home school	Child between 6 and 8 years old living in Colombia and is homeschooled

Out-of-school	An out-of-school child between 6 and 8 years old living in Colombia


### Procedure

A methodological matrix was developed (Table 2), given that the most widely used methodologies to inquire about coping and resilience processes are open interviews, observations, and reports from parents or teachers (Skinner & Zimmer, 2006). Particularly, we used interviews, observation of participants, and application of a specific instrument from BASIC Ph model. First, interviews were used to analyze family and individual characteristics before and after the pandemic, family structure, involvement of the family in the child’s education, and the economic and connectivity characteristics of the family. Interviews were also used to learn about the children’s resilience process, or the process perceived by their family.

**Table 2:** Methodological Matrix.


CONCEPT	CATEGORY	SUBCATEGORY	SOURCE

Context	Social and educational context	Family features	Interview with primary caregivers (parents)

		Pandemic experiences	

		Social and economic factors	

		Educational features	

	Human development	Corporal dimension	Participatory observation of the child

		Cognitive dimension	

		Social and emotional dimension	

		Communicative dimension	

Resilience and coping	Resilience	Family perception about child’s resilience	Interview with caregivers

		Observation of resilience promoting factors	Participatory observation of the child

	Coping	Belief channel	Six-part story measurement (6PSM) instrument from BASIC theory

		Affective channel	

		Social channel	

		Imagination channel	

		Cognitive channel	

		Physiological channel	


Second, participant observations were conducted to assess the physical, emotional, social, and cognitive development of each child. Third, the Six-Part Story Measurement (6PSM) model was employed to inquire about the resilience channels of the BASIC Ph model. Finally, we analyzed the similarities and differences between the case studies in relation to their corresponding educational contexts, coping channels, and resilience-promoting factors by using a comparison matrix.

#### First Contact, Ethical Considerations, and Preparations for Observations

Initially, communication was established with the families of each subject to share the action plan, establish agreements, analyze the social costs, and discuss the ethical issues involved. Also, issues mentioned by the caregiver with whom the first communication was held were considered as a first input for a given case.

Further, instruments were selected and designed as a system of gathering, recording, and classifying data. Other theoretical structures that could guide data collection were also analyzed, and plans were designed for the writing of the final report. Subsequently, the instruments were sent out for external review to identify and make any necessary adjustments.

In addition, an exhaustive review of the participants’ contexts was carried out to allow the researchers to obtain a better contextualization of each case before the observation process started. Likewise, a meeting was held with the participants and their caregivers to better understand their daily routines and to avoid affecting them negatively during the research. According to Hernández et al. (2019), this is an important element in obtaining a reflective stance and avoid influencing participants.

#### Data Collection and Validation

For this stage, questions and actions were planned beforehand to avoid inducing responses or behaviors in the participants, to prevent the researcher from assuming judgmental attitudes toward the responses, and to ensure several data sources were available for a sound triangulation process (Hernández et al., 2019).

First, a general observation of the family context of each participant was carried out. Then, for each case, a more detailed observation of the children, their caregivers, and the physical and human elements of their immediate context was completed. The observations were recorded in a format, whereby descriptive elements were written up using one color and interpretative elements were noted using another color, following Cuevas’ suggestion (as cited in Hernández et al., 2019).

Second, open-ended interviews were conducted with the primary caregivers; that is, a series of questions guided the conversation in relation to the objective of the research, but with flexibility to allow changes when needed. Notes were taken during the interview and this information was later organized to make it useful for analysis (Stake, 1998). The questions selected in the methodological matrix were restructured in accordance with the specifics of each case.

Third, the observation of each participant was adjusted to the characteristics of each case. This time, a participatory observation was conducted using a more specific instrument that included the units of analysis being observed, which corresponded to each of the dimensions of development. This observation was carried out using different games and cognitive, motor, social, moral, and artistic activities aimed at observing each of the dimensions.

Finally, an interview was conducted with each child, and the 6PSM instrument of the BASIC Ph model proposed by Lahad et al. (2013) was applied. This section of the study consisted of an activity divided into two parts: (a) an artistic creation based on a series of images and objects that reminded the children of the pandemic; and (b) a literary creation activity based on the BASIC Ph model (Figures 14, 15, 16, 17, 18, 19).

#### Data Analysis

While the instruments were being applied, a process of analysis began in order to explore the data, organize them into the categories of analysis initially proposed, describe participants’ experiences in narrative form, identify common patterns or elements between a case study and the situation surrounding it, understand the context of each case, reconstruct facts, and align the information collected with the theoretical framework of the study (Hernández et al., 2019).

During and after the field work, a detailed analysis of the information collected was carried out to reflect about the written material, audios, photographs, and videos, which were transcribed by using the qualitative analysis software ATLAS.ti (2022). An analysis log was developed after completion of the scanning for the purpose of understanding the units of analysis previously proposed, describing the new categories that appeared, and starting the process of grouping the information into categories and codes according to the themes, relationships, and patterns present in the information (Hernández et al., 2019).

Since the present research study corresponded to the nature of a collective case study or a comparative case study, a comparative matrix was construed (see Table 3) systematizing all the sources of each case to analyze similarities and differences and, thus, be able to explain the causes of the phenomena (Hernández, 2010).

**Table 3:** Comparative Matrix.


	PUBLIC CASE	PRIVATE CASE	RURAL CASE	MIGRANT CASE	HOMESCHOOL CASE	ETHNIC CASE

Social and economic context	Urban and low socioeconomical status in Bogotá. Lives in a single room with mother and sister.	Urban and medium socioeconomic status. Lives in Medellín in an apartment with mother, father, and brother.	Rural with difficult road access. Lives in Huila on a farm with mother, father, sister, and cousins.	Migrant from Venezuela that has been living in different areas of Colombia. Lives in Bogotá in a room with mother and father.	Urban and high socioeconomical status. Lives in grandfather’s apartment with father, mother, and sister. She travels continuously to her family’s farm.	Rural area near Bogotá inside an indigenous reservation. Lives in a house with mother, brothers, and mother’s husband.

Family context	Youngest son of a family with a mother as the head of household. He has an active relationship with his father.	Youngest son of a nuclear family. His brother is two years older.	Youngest daughter of a large nuclear family. She has cousins of her same age.	Single child from a nuclear family that lives away from their hometown.	Oldest daughter of a nuclear family that attends a Christian church.	Middle child from a family where the biological father is absent.

Educational context	Public school. During the pandemic, the school activities were developed at home and were sent by WhatsApp.	Current change of school from one private school to another private institution. During the pandemic, online classes were developed.	Rural school. During the pandemic, the school activities were sent by WhatsApp. At the moment there is no return to school yet.	Education developed at home due to difficulties with birth certificate for gaining access to public education at the country.	Education developed at home due to parents’ wish. Experimental education based on her interests and talents.	Public ethnic school. During the pandemic, the school activities were developed at home and were sent by WhatsApp.

Difficulties in the pandemic	Difficult tasks sent by school and internet access.	Paying attention during online classes.	Having no access to school after the lockdown.	Not been able to travel to visit family.	Not been able to travel to visit animals at the farm.	Not been able to go outside the house to play with cousins.

Stress reactions	Cry	Get distracted playing	Cry	Walk away	Bite nails	Cry

Resilience promoting factors	Problem solving, previous difficulties, and family support.	Problem solving, sense of humor, and creativity.	Self-esteem, family support, and sense of belonging with school.	Problem solving, introspection, previous difficulties, and initiative.	Creativity, family support, religious beliefs, and initiative.	Problem solving, initiative, indigenous community support, and self-esteem.

Coping channels	Physiological and Cognitive Channels	Cognitive Channel	Physiological and Benevolence Channels	Social Channel	Physiological Channel	Physiological Channel


## Results

The data collection instruments were applied one case at a time. It should be noted that the selection of the cases was the result of connections with educational institutions, teachers, and acquaintances in different territories of Colombia and that the results demonstrate a range of social, economic, educational, family, and personal contexts (Table 3).

As mentioned, to describe the resilience processes experienced by each of the participating children, multiple data sources were considered, including the perceptions of the primary caregivers and the views of the participants themselves. Throughout the data collection process, the information was double-checked in order to confirm that the information collected had been understood correctly by the researcher. Although the results of the study are not generalizable, the instruments and procedures can easily be replicated in other national and international contexts, with some minor adjustments. Before presenting the results, each case study will be briefly described.

### Description of Participants (Names Are Fictitious)

#### Santiago: Public-School Case

**Figure 2:**
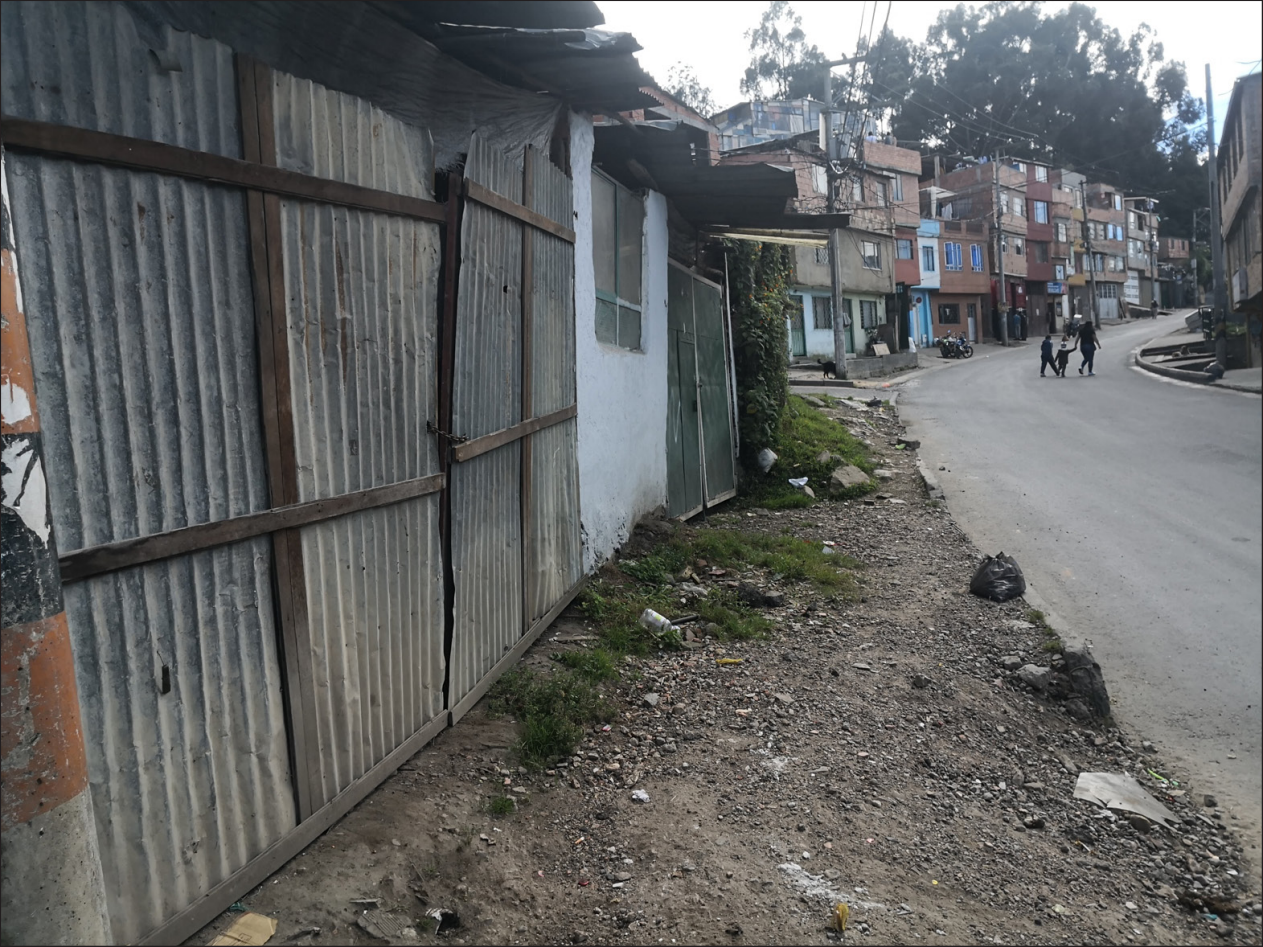
Santiago’s Neighbourhood.

**Figure 3:**
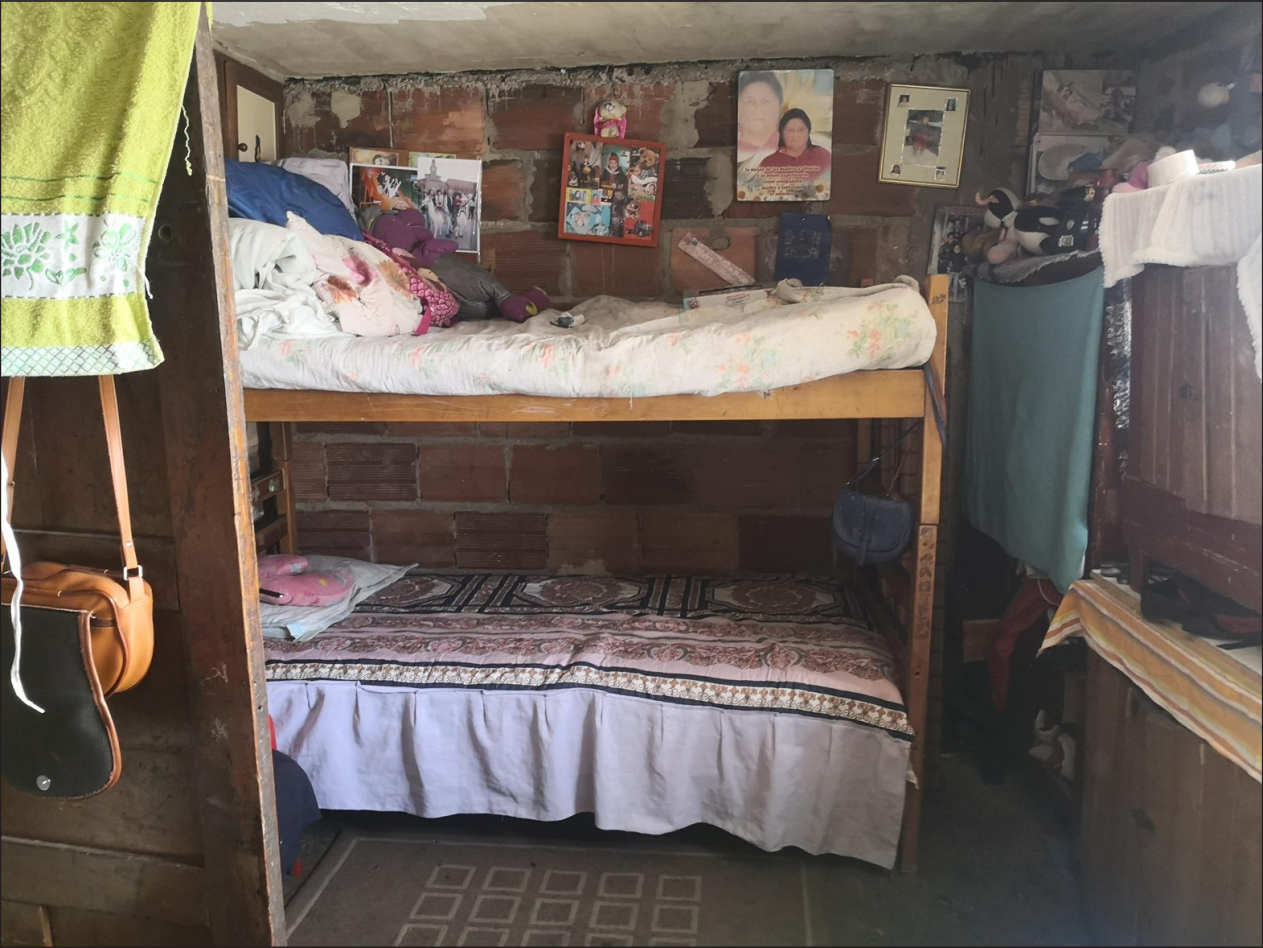
Santiago’s Family Room.

Santiago is a 7-year-old boy who lives in a low-income neighborhood in Bogotá (Figure 2), the capital of Colombia. He lives with his mother and sister in the same room in a house that they share with other family members (Figure 3). When the COVID-19 pandemic broke out, Santiago’s mom could not have a birthday party for him and his friends as usual. This made Santiago feel sad and lonely, and this feeling was present throughout the months of the pandemic, also because his mother had to seek part-time jobs to feed the family.

#### Tomás: Private-School Case

**Figure 4:**
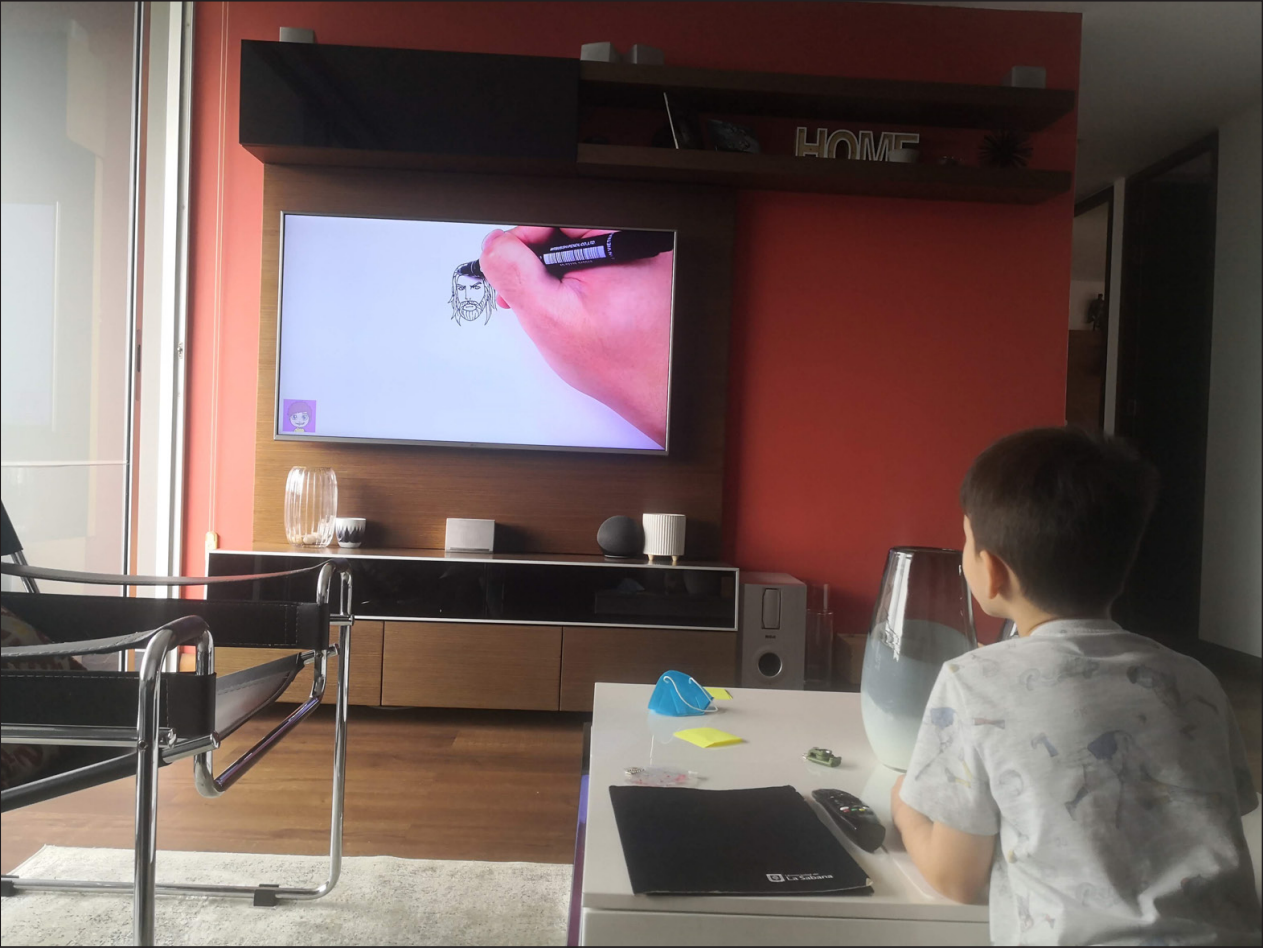
Tomás’s Online Lessons.

**Figure 5:**
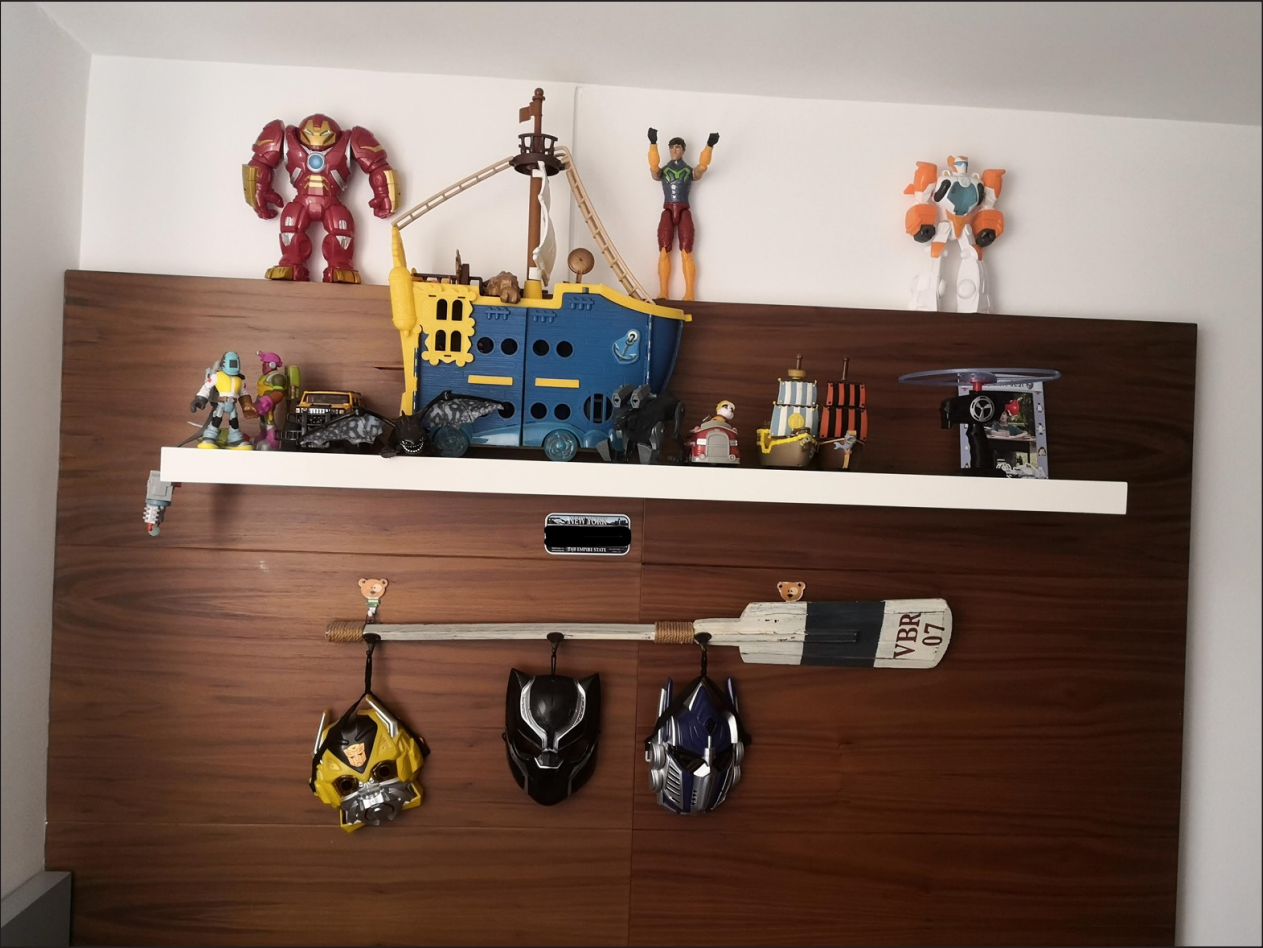
Tomás’s Toys.

Tomás is a 7-year-old boy who had just entered a new school in Medellín when the pandemic broke out. During the pandemic, he was 5. Tomás received online classes all day (Figure 4). That was difficult for him and his medium-income family because his attention span was short. After some months, his parents decided to quit online education for their son and look for a tutor. Tomás does not like school, he prefers to stay home playing with his brother and their toys (Figure 5).

#### Lucía: Rural Case

**Figure 6:**
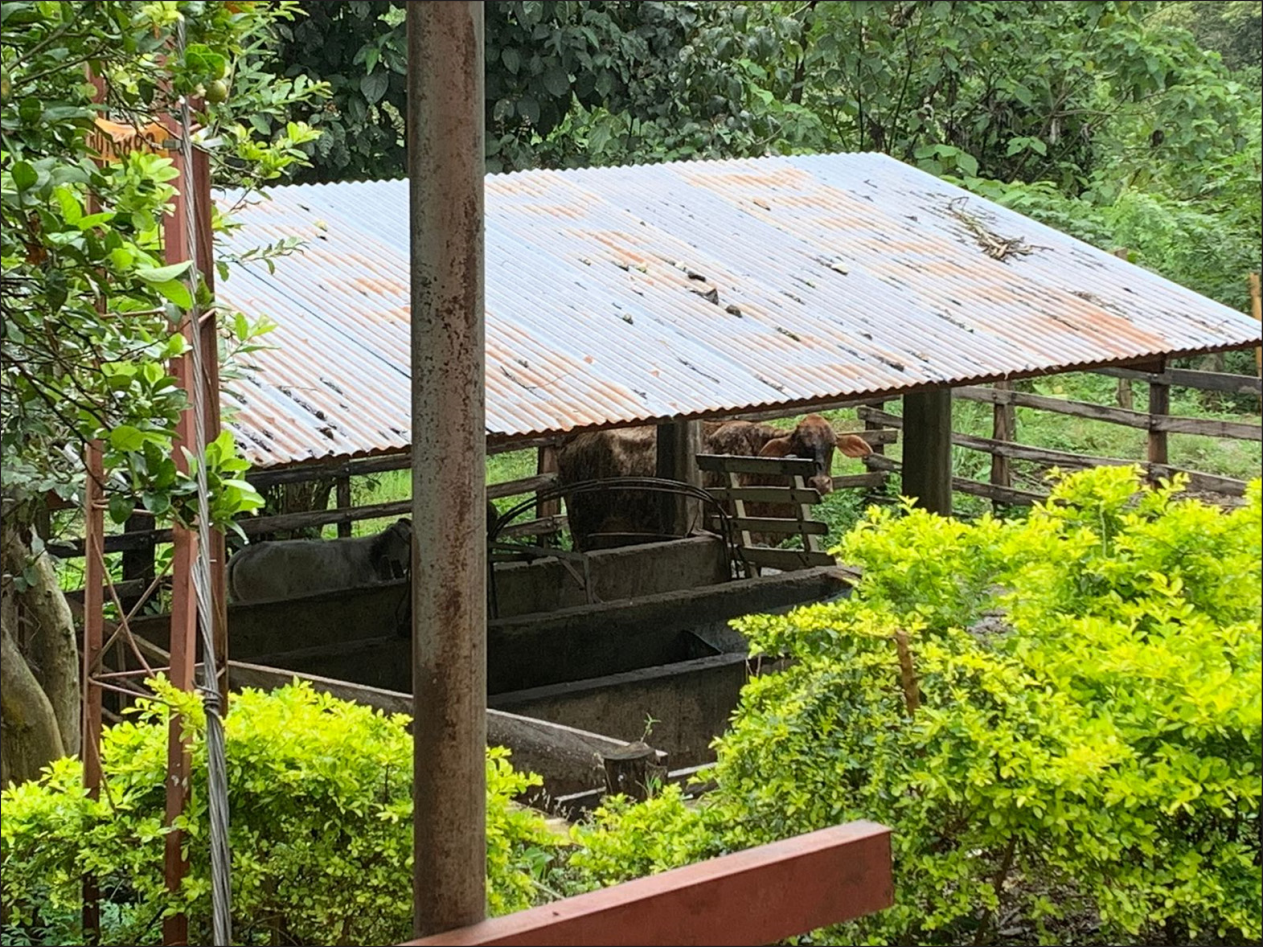
Lucía’s Farm.

**Figure 7:**
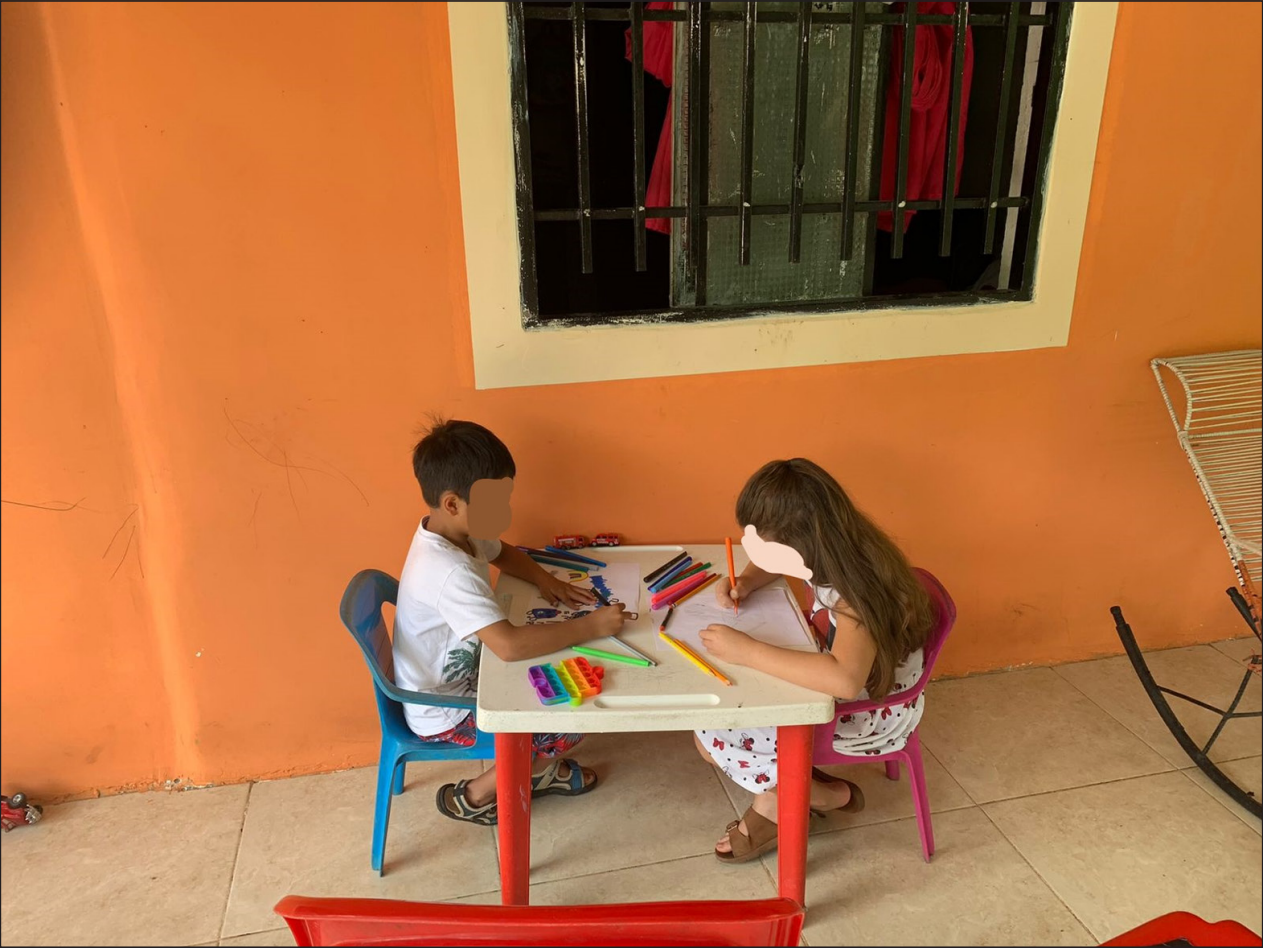
Lucía and Her Cousin.

Lucía is a 6-year-old girl who lives in a rural area in Huila, in southwest Colombia (Figure 6). She lives on a farm full of animals, surrounded by nature and water, along with parents, sisters, and cousins (Figure 7). At the time of the study, in 2022, Lucía still could not go to school, because her school does not have enough teachers for all the students. Lucía felt happy during the COVID-19 pandemic because she could play with her family and help them by doing the chores.

#### Daniel: Immigrant Case

**Figure 8:**
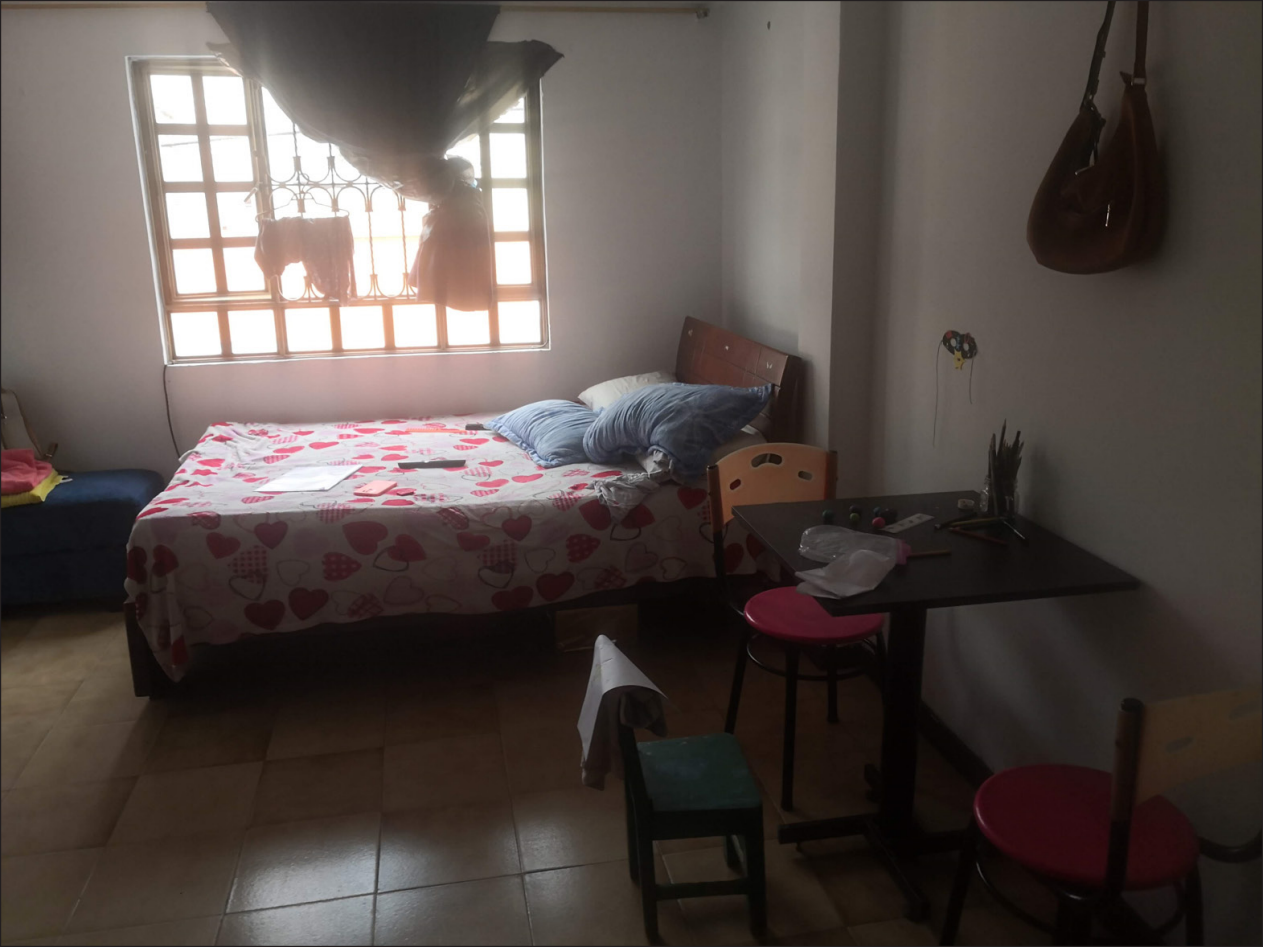
Daniel’s Room.

**Figure 9:**
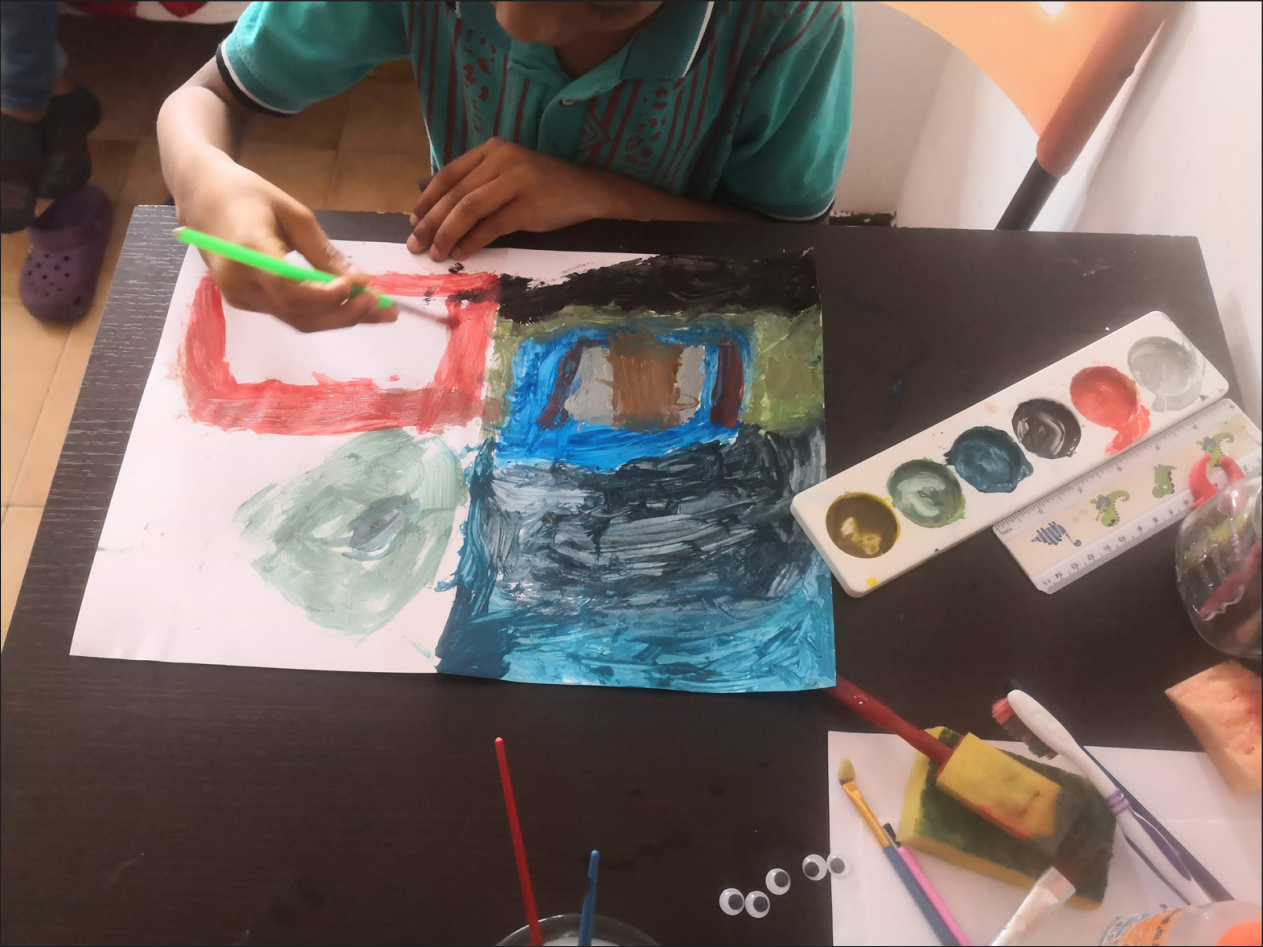
Daniel Painting His Grandfather’s House.

Daniel is an 8-year-old boy who lives in Bogotá with his parents (Figure 8). Before settling there, he had moved several times and lived in his country of origin, Venezuela. Because of the economic crisis in his country, he came to live in Colombia. Daniel’s greatest dream is going to school; every time he sees a boy or girl in a school uniform, he feels sad. Due to administrative problems with his birth certificate, he falls outside the educational services in Colombia and has not been in a school since he was 3 years old. In addition, during the COVID-19 pandemic Daniel lost his grandfather, who belonged to an ethnic community in Colombia (Figure 9).

#### Julieta: Homeschooling Case

**Figure 10:**
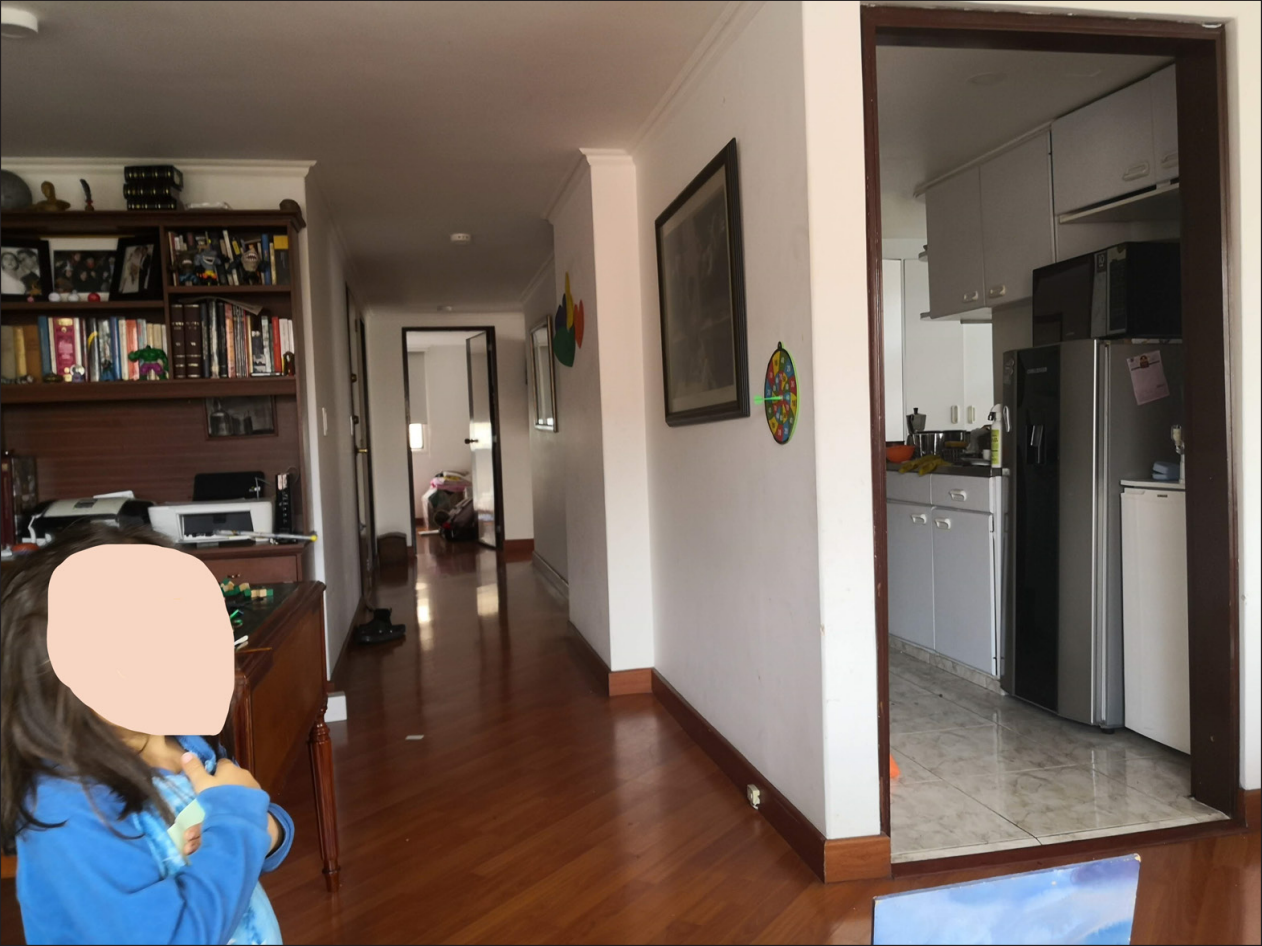
Julieta’s Apartment.

**Figure 11:**
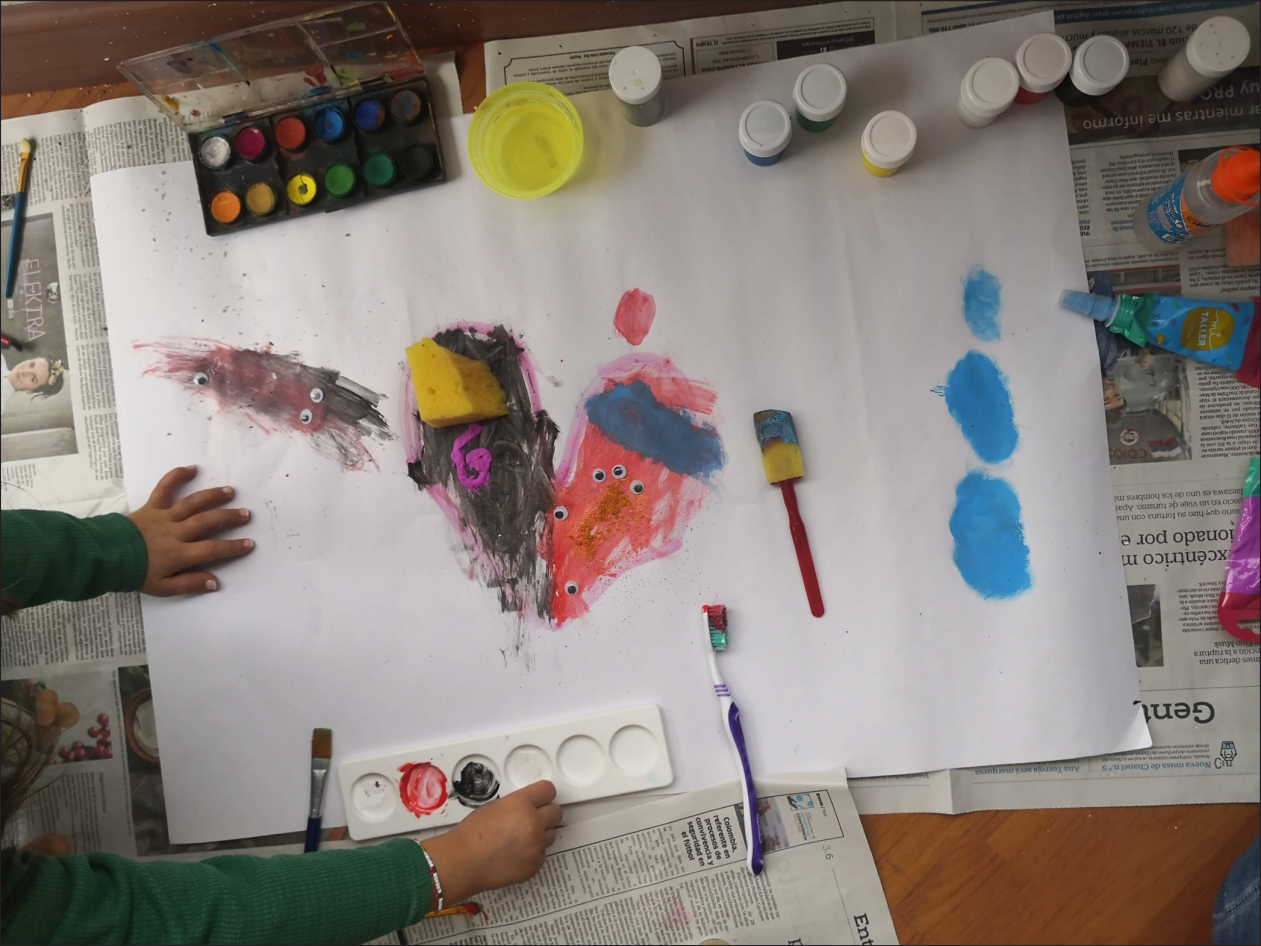
The Pandemic According to Julieta.

Julieta is an 8-year-old girl who lives in an apartment in Bogotá with her parents and sister (Figure 10). Although the study took place in the middle of the school year, the family just arrived from a long trip to France and the United States. For Julieta’s family, education occurs in everyday home experiences, not at school. For this reason, all Julieta’s learning takes place at home directed by her mother. The COVID-19 pandemic was difficult for Julieta because, during that time she couldn’t do her duties on the family’s farm, which is located near the city, and where she takes care of different animals. During that time, Julieta started to bite her nails (Figure 11).

#### David: Ethnic Case

**Figure 12:**
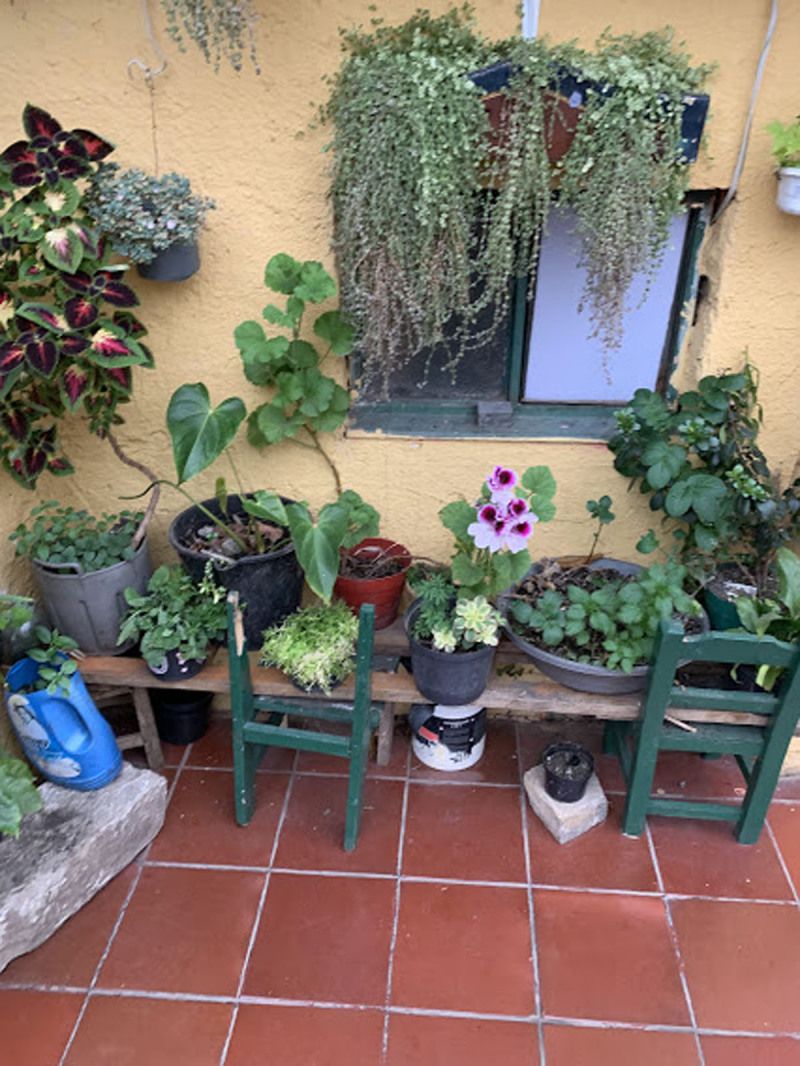
David’s Home.

**Figure 13:**
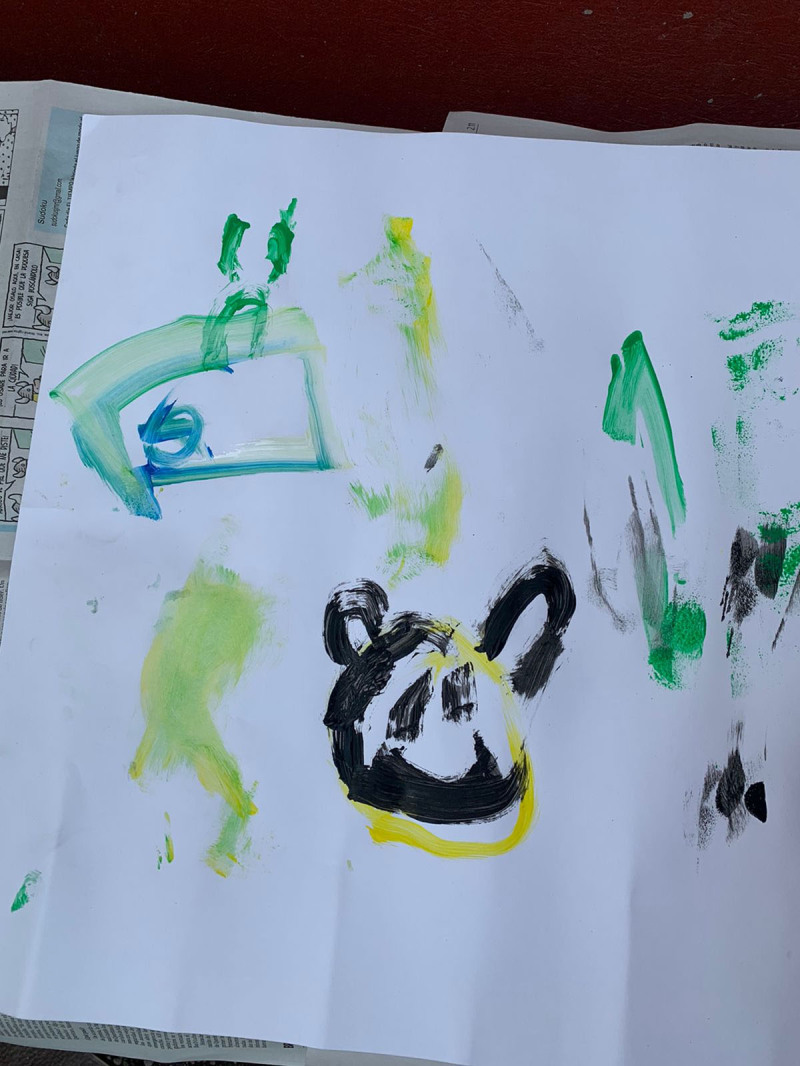
The Pandemic According to David.

David is a 6-year-old-boy who lives on an indigenous reservation near Bogotá, in a house with his mother, brothers, and his mother’s husband (Figure 12). David goes to a special public school run by his indigenous community, where he learns about his culture and language. During the pandemic, the people in the community helped each other; for David, not being able to go outside to play with his cousins was difficult and made him feel sad (Figure 13).

**Figure 14:**
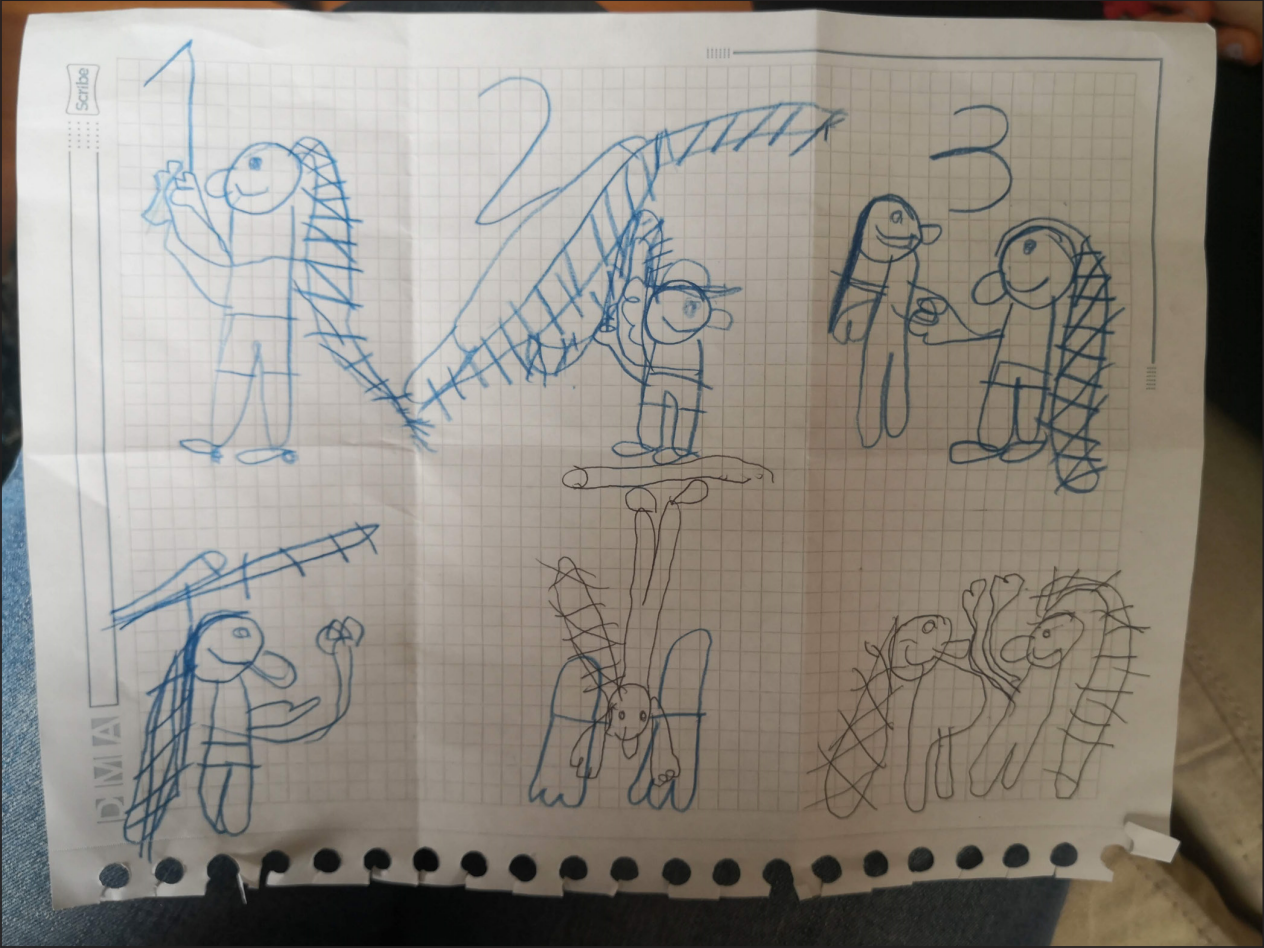
Julieta’s 6PSM.

**Figure 15:**
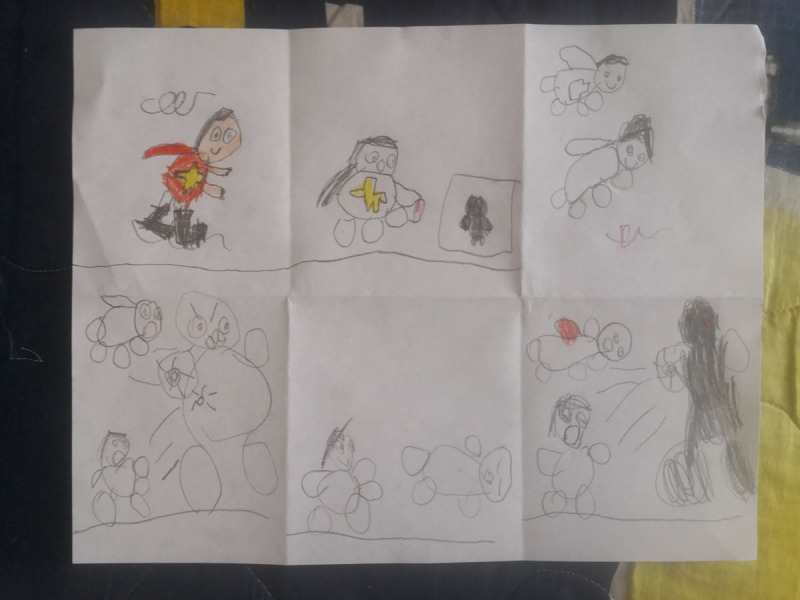
Daniel’s 6PSM.

**Figure 16:**
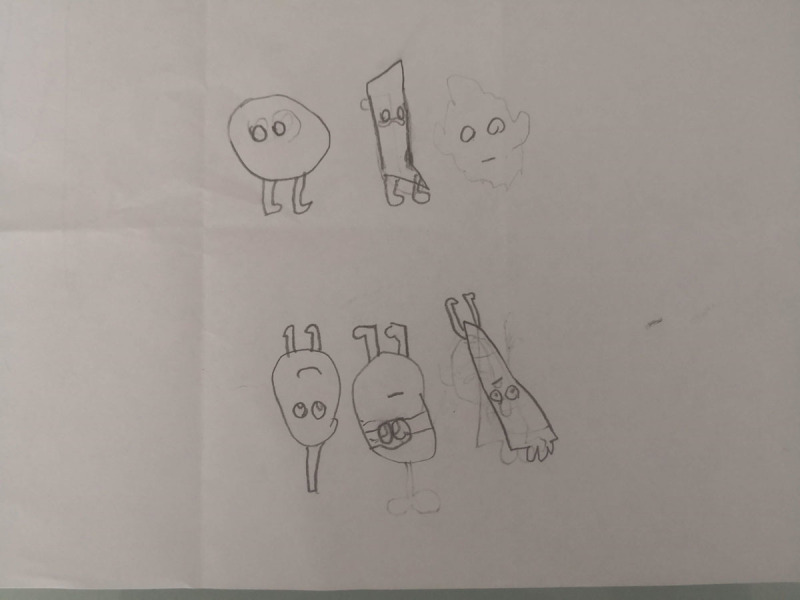
Tomás’s 6PSM.

**Figure 17:**
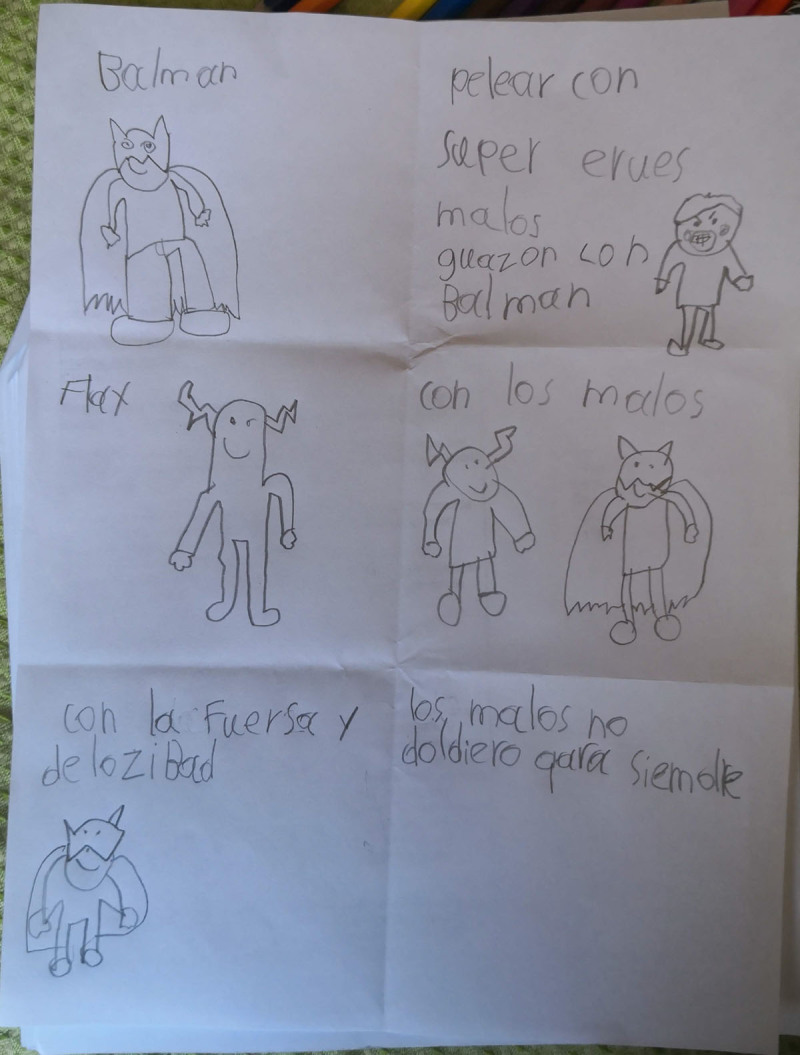
Santiago’s 6PSM.

### Family, Community, and Educational Factors

An analysis of all the cases showed that the participating children had at least one support network before and throughout the COVID-19 pandemic. In all cases, the predominant support network was the family, as evidenced in the support statements; affection and care mentioned in the interviews with the main caregivers (mother and father or only mother) and the record of observation of participants showed no evidence of child neglect.

Likewise, in some cases, specifically the children attending public schools, parents reported that the relationship the children had with the school (current or former) and with their teachers was positive. This is fundamental for the development of resilience, because – as mentioned in the theoretical framework – teachers are resilience tutors for children living under complex socioeconomic and family conditions (Cyrulnik, 2018).

Two of the participating families belonged to an indigenous community. During the pandemic, despite the inequality they experienced and the poverty and discrimination present in these communities, they were able to find solutions to their challenges through community support and traditional health practices (Ortiz, 2020). Also, one of the families was part of a religious community, a fact that may have influenced the development of resilience, as it has been found that people with religious beliefs have better coping and adaptation skills when faced with adversity (Román et al., 2020).

### Individual Factors

Initiative, cognitive flexibility, problem solving, and strategic thinking are part of executive functioning, and are skills that increase the levels of resilience and social cognition (Losada & Quijano, 2017). We were able to identify elements of these skills by using the instruments applied with the participating children,

Initiative, understood as the ability to start an activity without being asked, that is, to suggest ideas, answers, or solve problems independently (Soprano, 2003) was observed in some children’s behaviors, such as the ability to propose new activities or actions to complement the activities initially proposed. For example, proposing to build new obstacles in the treasure hunt or proposing to create sculptures or symbolic games to represent the story created from the 6PSM.

Another skills, cognitive or emotional flexibility, which is a component of future-oriented competence (Feder, 2018), was observed in the children’s response to the constant changes in the proposed activities. Likewise, problem solving was observed with all the instruments, especially in those moments when the children had to respond to hypothetical situations, create something or identify sensations with all their senses. For example, when the children had to identify a certain number of sensations that they could see, hear, smell, touch, and taste; some children resorted to creative solutions such as activating virtual assistants by voice to hear a sound or washing their hands so that they could smell something specific.

Further, problem-solving capacity was observed in the game of marbles that required children to identify the options and make decisions accordingly in order to win the game. Examples include giving marbles to the other player when there were few marbles on the board but putting them on the board instead of giving them when the player had enough marbles in order to increase the possibility that the opposing player had more marbles, or even trying to trick the other player by changing the outcome of the throw of the dice.

The action just mentioned is related to morality, recognizing that ethics and morality are integrated into the development of resilience, since resilient persons have ethical competencies that enable them to live in society and act in favor of moral wellbeing (Carreño, 2009). In the cases analyzed, some children showed an ability to develop empathy and social perspective to solve hypothetical conflicts. For example, when faced with the hypothetical situation of breaking something of family value while playing with another person, several children responded that they would accept that they were partly to blame for what happened and would repair their mistake by apologizing.

Other pillars of resilience also determine whether a person, in this case a child, is likely to develop positive coping. Some of these pillars include creativity, a sense of humor, introspection, independence, and relational skills (Wolin, as cited in Melillo et al., 2002). Creativity was evidenced in the answers that the participating children gave to the proposed challenges; the sense of humor, which was present especially in one of the cases, is a characteristic that allows reconfiguring a situation to change behavior (Melillo & Suárez, 2002). Finally, introspection, independence, and the ability to relate to other people were evidenced in some cases more than in others.

### Coping Channels of the BASIC Ph Model

**Figure 18:**
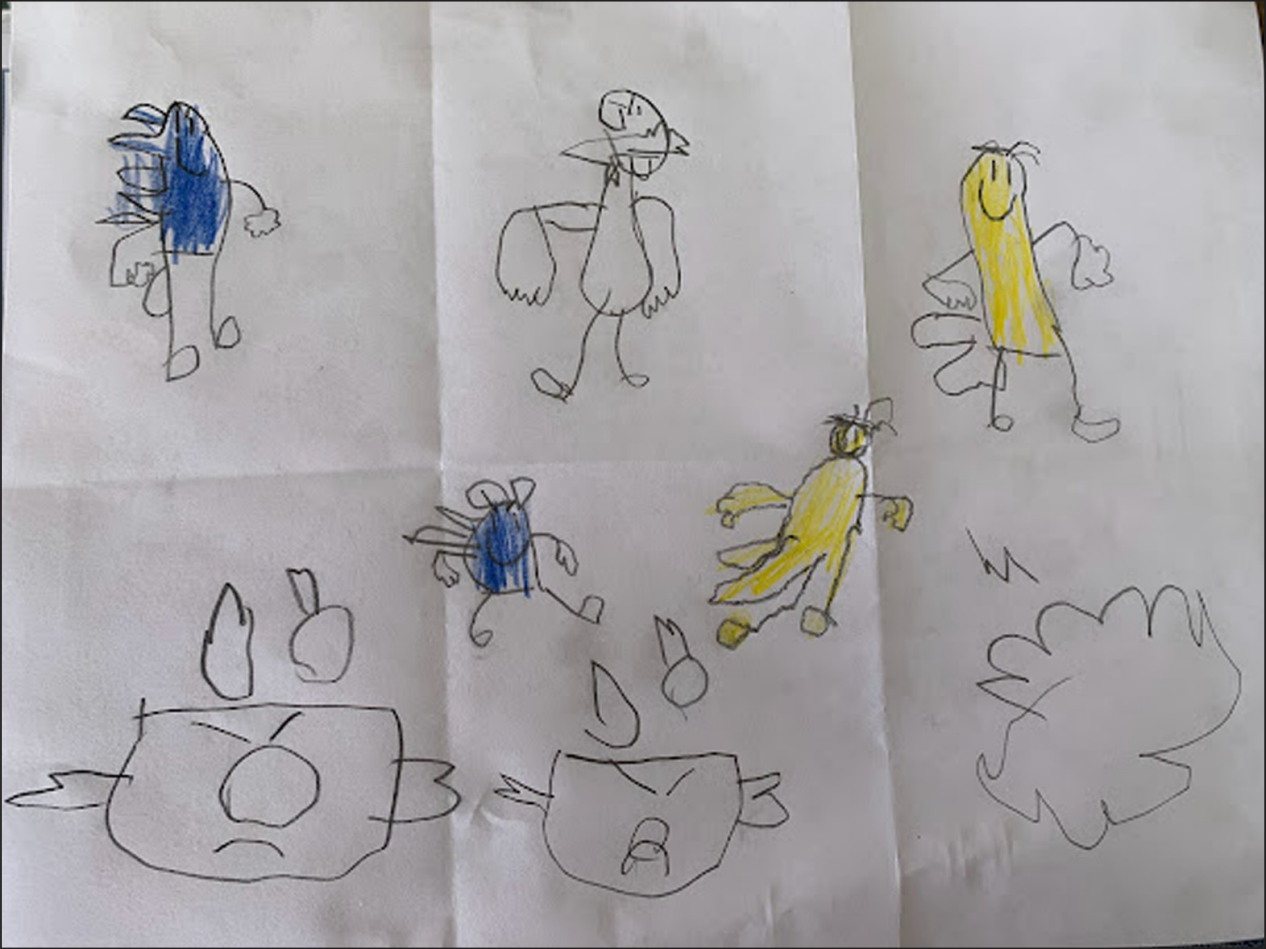
David’s 6PSM.

**Figure 19:**
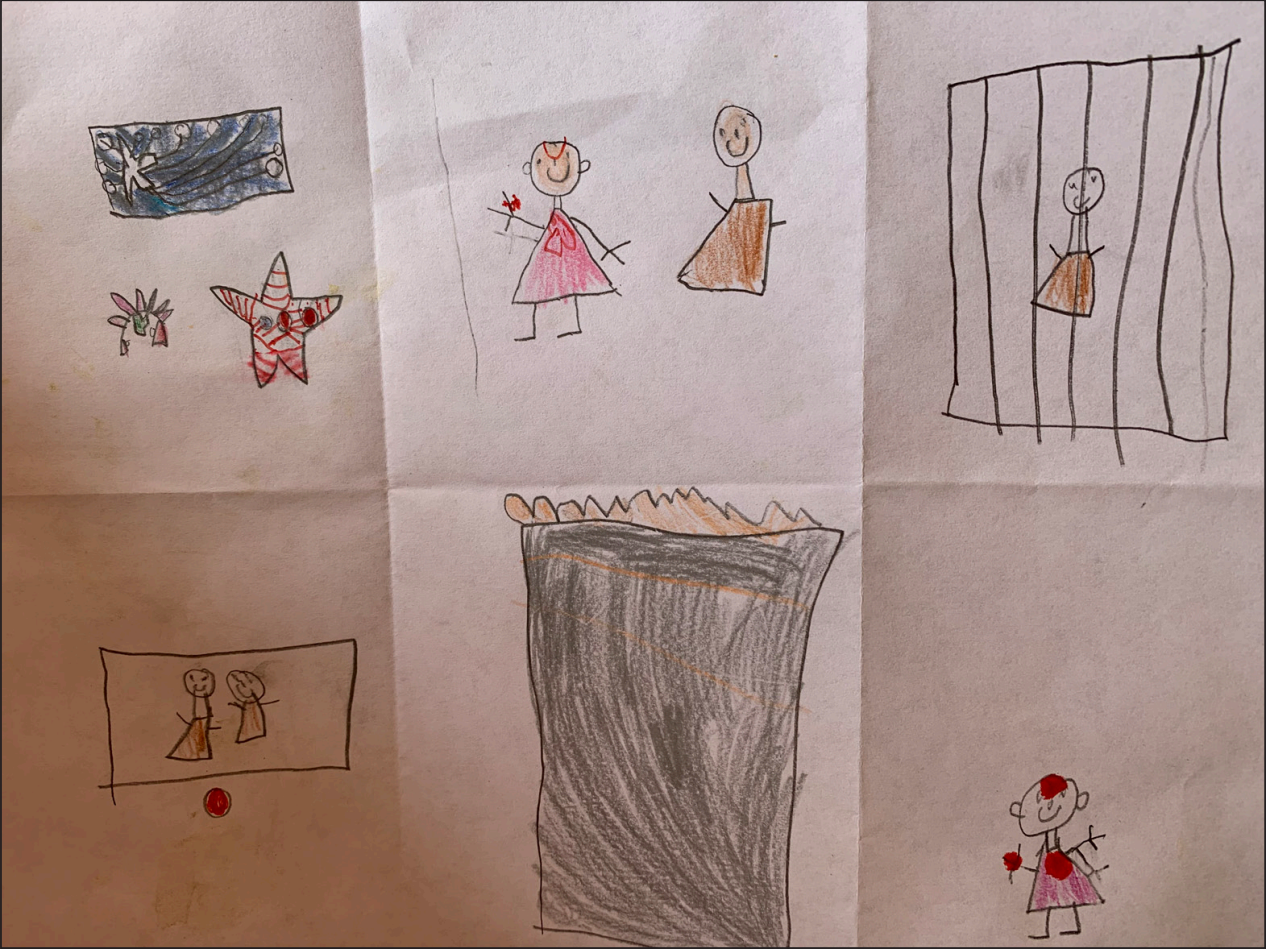
Lucia’s 6PSM.

The coping channels were identified with the 6PSM instrument proposed by Lahad et al. (2013) (Figures 18–19). Consequently, the presence or absence of the different coping channels in the narrative of each of the participating children was analyzed based on the theory of the BASIC Ph model. Some examples of the stories obtained and the segments that correspond to a specific coping channel are presented below.

First, the benevolence channel is related to the ability to build a personal identity from moral, ideal, or religious values (Shacham & Ayalon, 2013). The segments of the stories that correspond to this channel respond to moral labels such as “good,” “bad,” “evil,” “villain,” “robbery,” and “jail.”

Second, the affective channel encompasses the perception, analysis, and management of emotions and their triggering behaviors or symptoms (Lahad et al., 2013). Although the participating children mentioned emotions such as sadness, fear, anger, and happiness in response to specific questions, none of the accounts included concepts corresponding to this channel.

Third, the social channel responds to elements of the stories that include more than one character, such as the companions of the superhero, protagonists, or helpers, referred to by some children as “friends,” “policemen” or using the name of a family member or well-known superhero. Similarly, stories that included more than one villain or a group of people who were saved were assigned to this coping channel.

Fourth, the imaginative channel was associated with sections of stories that related to out-of-the-ordinary characters and situations, such as food-shaped characters, cowboys living inside boots, and unusual powers, such as throwing diamonds.

Fifth, the cognitive channel encompasses all text segments that talk about cognitive reasoning of the characters or events in the story. Some examples are “they did it because they wanted to take it over,” “but the policeman took it out again because he thought it was good,” “it had the power to convince the bad guys of something,” and “they had left ashes where they were going to attack.”

Finally, the motor or physiological channel includes all bodily elements, both body parts and actions that can be performed with the body. This channel included attributes of the protagonists such as “strength” and “speed,” as well as actions such as “make a hole with the ring,” “send the net,” “throw very far and freeze close,” “handstand,” and “tie with long hair.”

## Discussion

The results of this research study illustrate the development of coping and resilience processes during the COVID-19 pandemic of children from different geographical and educational contexts in Colombia. These findings not only help to gain a better understanding of the different circumstances experienced by the children during the pandemic, they also help to emphasize the importance of the developing resilience-promoting factors and coping channels in the educational system, specifically in childhood.

The most predominant resilience-promoting factors included problem solving, a sense of humor, initiative, creativity, self-esteem, family support, facing difficult situations, and social support from a community. These factors are in line with the results of other studies carried out in Latin-America that found that humor, self-esteem (Salgado, 2005), introspection, initiative, creativity (Melillo, 2002, as cited in Duarte, 2016), and community and family support (Amar et al., 2003) were resilience-promoting factors.

In addition, caregivers, in some cases both parents and in other cases only the mother, highlighted behaviors that their children usually exhibit when facing a difficult situation. In most cases, crying was the most frequent behavior, a response that corresponds to the Affective coping channel (AC), and that was identified as the least predominant channel in the results of the 6PSM instrument; it also belongs to the physiological channel (PhC), as it represents a bodily action. Similarly, nail-biting is also included in the physiological channel, since it involves a non-positive action involving the body but due to an emotion (i.e., as part of the Affective coping channel).

This is how the BASIC Ph resilience and coping model identifies the coping channels most used by the person, in this case children, but at the same time it shows the less present channels to strengthen them to develop resilience (Lahad, 2016). Therefore, the affective channel presents a paradox in this research, as it is absent in the narrative of all participants, but present in the testimonies of their parents and in the participant observations. Based on these findings, it can be inferred that although the participating children naturally express their emotions through bodily actions, such as crying or nail-biting, it is not a coping channel that they use consciously. For this reason, it is essential to continue strengthening the recognition and management of emotions in educational settings.

Interesting, the parents of the private and the migrant cases emphasized that their children demonstrated different behaviors when facing a problem situation. In the first case, the child tended to distract himself with games or jokes, something that responds to the cognitive coping channel predominant in the results his 6PSM story. In the second case, the child reacted to his grandfather’s death by asking questions and distancing himself from his cousins, something that responds to the social coping channel, since help-seeking and social isolation are behaviors typical of this channel.

With respect to the migrant case, his predominant coping channel may respond to his need to socialize with peers since, in addition to the fact that he is an only child, at various times during the pandemic the child expressed an interest in going to school and being reunited with his extended family. This differs from the homeschooling case since, although the child receives education at home, her parents emphasized in the interview that socialization with her sister, grandparents, neighbors, and friends is a fundamental pillar in her formative process.

In summary, various types of coping were evidenced in the children during the COVID-19 pandemic, some of them negative, such as nail-biting, and others positive, such as inventing games to distract themselves, expressing their emotions, and seeking support from others. However, it is difficult to determine whether the participating children developed resilience during this time. Although it is known that positive coping leads to adaptation to the situation (Kumpfer, 1999), the development of resilience starts in a traumatic situation, something that the pandemic may not have represented for the participating children.

Indeed, based on the findings of this study, it is clear that all participating children had internal resilience-promoting factors and will be able to strengthen their coping channels and develop resilience in the future, provided a stable family support network and a comprehensive educational process that fosters the development of all dimensions of their personalities.

## Conclusion

The children who participated in this study had to face difficult situations during the pandemic, each of them responding with one or two predominant coping channels determined by their personality, experiences, and competencies. However, it is important to note that the educational process of each child strengthened the other coping channels, giving them more tools to deal with stress. Resilience leads to the construction of sustainability and well-being at a national and global level because, based on individual actions such as resilience-promoting factors and coping channels, complex situations may be overcome without affecting oneself or the surrounding environment.

The relationship found between the results of the 6PSM instrument and the information obtained from participant observations and interviews with the parents confirms that this was a valuable instrument that may be applied in other areas beyond psychology to identify people’s coping channels.

Additionally, common elements were identified among the cases that did not apply directly to this study, but that are of interest for further study. For example, one of these relates to the importance that children who live in rural areas place on technology, since both rural cases studied here (rural case and ethnic case) mentioned television or video games as things they like to do in their free time, while children in urban areas mentioned playing, reading, and contact with nature.

Also, since this was a case study, it was small in scope, so it would be interesting to replicate it with a larger number of participants. Furthermore, extending the study to an international context would allow educators to understand the relationship between the case studied and the community in different types of state-run organizations, shedding light on the importance of the role of the individual and the role of the community during the pandemic. Along with these limitations, another topic of interest in future studies is the use of space and colors in the children’s drawings.

In conclusion, the results of this investigation yield valuable information to educators, psychologists, social workers, and other stakeholders about the different experiences, coping processes, and learnings that children from different contexts were exposed to during the COVID-19 pandemic.

Similarities exist between the findings of this study and others conducted in Colombia. In the present study, as in most of the others, the need for further research about the relationship between comprehensive child development and the presence of resilience-promoting factors is highlighted. Finally, although limited, we feel that the existing body of research h provides a solid basis for training members of children’s support networks to be prepared for situations like the COVID-19 pandemic in the future.
